# ERCC1 Is a Predictor of Anthracycline Resistance and Taxane Sensitivity in Early Stage or Locally Advanced Breast Cancers

**DOI:** 10.3390/cancers11081149

**Published:** 2019-08-10

**Authors:** Tarek M. A. Abdel-Fatah, Reem Ali, Maaz Sadiq, Paul M. Moseley, Katia A. Mesquita, Graham Ball, Andrew R. Green, Emad A. Rakha, Stephen Y. T. Chan, Srinivasan Madhusudan

**Affiliations:** 1 Department of Oncology, Nottingham University Hospitals, Nottingham NG5 1PB, UK; 2 Translational Oncology, Nottingham Breast Cancer Research Centre, Division of Cancer and Stem Cells, School of Medicine, University of Nottingham, Nottingham NG5 1PB, UK; 3 School of Science and Technology, Nottingham Trent University, Clifton Campus, Nottingham NG11 8NS, UK; 4 Academic Pathology, Nottingham Breast Cancer Research Centre, Division of Cancer and Stem Cells, School of Medicine, University of Nottingham, Nottingham NG5 1PB, UK

**Keywords:** ERCC1, anthracycline resistance, taxane sensitivity

## Abstract

Genomic instability could be a beneficial predictor for anthracycline or taxane chemotherapy. We interrogated 188 DNA repair genes in the METABRIC cohort (*n* = 1980) to identify genes that influence overall survival (OS). We then evaluated the clinicopathological significance of ERCC1 in early stage breast cancer (BC) (mRNA expression (*n* = 4640) and protein level, *n* = 1650 (test set), and *n* = 252 (validation)) and in locally advanced BC (LABC) (mRNA expression, test set (*n* = 2340) and validation (TOP clinical trial cohort, *n* = 120); and protein level (*n* = 120)). In the multivariate model, ERCC1 was independently associated with OS in the METABRIC cohort. In ER+ tumours, low *ERCC1* transcript or protein level was associated with increased distant relapse risk (DRR). In ER−tumours, low *ERCC1* transcript or protein level was linked to decreased DRR, especially in patients who received anthracycline chemotherapy. In LABC patients who received neoadjuvant anthracycline, low *ERCC1* transcript was associated with higher pCR (pathological complete response) and decreased DRR. However, in patients with ER−tumours who received additional neoadjuvant taxane, high *ERCC1* transcript was associated with a higher pCR and decreased DRR. High *ERCC1* transcript was also linked to decreased DRR in ER+ LABC that received additional neoadjuvant taxane. ERCC1 based stratification is an attractive strategy for breast cancers.

## 1. Introduction

Anthracycline and taxane based adjuvant and neoadjuvant chemotherapies are standard approaches in the management of early stage or locally advanced breast cancers to reduce distant recurrence and improve survival [1,2,3]. Moreover, the recent development of multi-parameter gene-expression assays, largely based on proliferation biomarkers, has facilitated the selection of patients who are most likely to benefit from systemic chemotherapy [4]. However, despite the genomic based selection, not all patients benefit from chemotherapy. In addition, chemotherapy related toxicity (such as anthracycline induced cardiotoxicity/leukaemia and taxol induced irreversible peripheral neuropathy) can adversely impact overall outcomes. Therefore, the development of anthracycline and/or taxane specific predictive biomarkers is desirable. 

Chromosomal instability (CIN) that alters chromosome number or structure is a hallmark of cancer including breast tumours [5,6,7]. Whilst genomic instability is a key driver of CIN, dysfunctional mitotic mechanisms, such as defective spindle assembly checkpoints and defective sister chromatid cohesions can also promote chromosomal instability [5,6,7]. Tumours with impaired DNA repair capacity and CIN are sensitive to DNA damaging chemotherapeutics. In early breast cancer patients, duplication of chromosome 17 centromere enumeration probe (Ch17CEP), a CIN marker, was previously shown to be a strong predictor of benefit from anthracycline adjuvant chemotherapy in a prospective clinical trial [8]. On the other hand, in a meta-analyses by the early breast cancer trialists collaborative group (EBCTCG), benefit from taxanes (paclitaxel, docetaxel) based chemotherapy was most evident in chromosomally stable low grade breast cancers [9,10]. In addition, CIN has been shown previously to predict paclitaxel resistance in ovarian cancer patients [11].

Chemotherapy induced or radiotherapy induced DNA damage is processed by various DNA repair pathways in cells. Emerging data provides strong evidence that overexpression of DNA repair factors can also contribute to therapeutic resistance in cancers [12]. DNA adducts induced by chemotherapy (such as by platinum and cyclophosphamide) are processed through the nucleotide excision repair (NER) pathway. NER is a highly conserved, versatile and robust. NER is a complex pathway requiring several proteins and their interacting partners. Although complex, two sub-pathways of NER have been described: The transcription-coupled nucleotide excision repair (TC-NER) pathway, that targets lesions specifically in the transcribed strand of expressed genes, and the global genome nucleotide excision repair (GG-NER) pathway, that deals with lesions in the rest of the genome. Although these NER sub-pathways are complex, basic steps in GG-NER include DNA damage recognition (by XPC-HR23B complex), lesion demarcation and verification by TFIIH complex (Cdk7, Cyclin H, MAT1, XPB, XPD, p34, p44, p52 and p62), assembly of a pre-incision complex (RPA, XPA and XPG), DNA opening by XPB and XPD helicases, dual incision (by ERCC1–XPF and XPG endonucleases), release of the excised oligomer and finally repair synthesis to fill in the resulting gap (RPA, RFC, PCNA, Pol δ/ε, and ligation by ligase I) [13,14]. In TC-NER, translocating RNA polymerase II detects lesions in the template. A role for ERCC8 (CSA) and ERCC6 (CSB) has also been suggested in DNA damage recognition in TC-NER. Subsequent steps in TC-NER are similar to GC-NER.

ERCC1 protein is a critical player in NER. ERCC1 is non-catalytic but associates with XPF endonuclease (also known as ERCC4) to form the ERCC1–XPF heterodimer. The ERCC1–XPF heterodimer cleaves and facilitates the removal of bulky lesions, such as those induced by platinum chemotherapy [15,16]. In addition the ERCC1–XPF heterodimer also has essential roles in other DNA repair pathways, such as DNA recombinational repair and inter-strand crosslink repair [17,18]. ERCC1 and XPF siRNA depletion was previously shown to increase cisplatin sensitivity in non-small lung [19] and breast cancer cells [20]. In a mouse xenograph model, ERCC1-deficient melanoma cells were also observed to be 10-fold more sensitive to cisplatin than ERCC1-proficient cells [21]. ERCC1 as a marker of chemotherapy resistance has been well described in other solid tumours, including lung, colorectal, head, neck, gastric, bladder and ovarian cancers [22,23,24,25,26]. Given the critical role of ERCC1 in genomic integrity, in the current study, we evaluated the role of ERCC1 as a biomarker in breast cancers.

## 2. Results

### 2.1. ERCC1 Transcript Is a Predictor of Tumour Grade and Chromosomal Instability in Early Stage breast cancers (BCs)

A large body of clinical evidence confirms that high grade BCs is associated with chromosomal instability. Given the critical role of ERCC1 in NER, DSB repair, ICL repair and chromosomal stability, we evaluated *ERCC1* transcripts in the METABRIC Cohort. A low level of *ERCC1* transcript was significantly associated with higher grade cancer, whereas low grade tumours were common in high *ERCC1* tumours (Table 1) (*p* values < 0.0001). Low *ERCC1* transcript was also associated with Ki67 positivity, ER−, PAM50 Luminal B, Pam50 Her2, Pam50 basal and Genufu ER+/HER− (high proliferation) tumours. On the other hand, low grade, Ki67 negative, PAM50 Luminal A and Genufu ER+/HER− (low proliferation) were more common in tumours with high *ERCC1* transcript (all *p* values < 0.0001). To evaluate associations with chromosomal stability we investigated ERCC1 in various integrative molecular cluster (intClust) phenotypes described in the METABRIC cohort. Low *ERCC1* transcript was linked to genomically unstable intClust.10 phenotype whereas high *ERCC1* was associated with chromosomally stable intClust.3, 4, 7 and 8 tumours. Together, the data provides the first clinical evidence that low ERCC1 is a marker of chromosomal instability and aggressive phenotype in BCs.

### 2.2. ERCC1 Transcript and Clinical Outcomes in Patients Receiving Adjuvant Therapy

**Figure 1:**
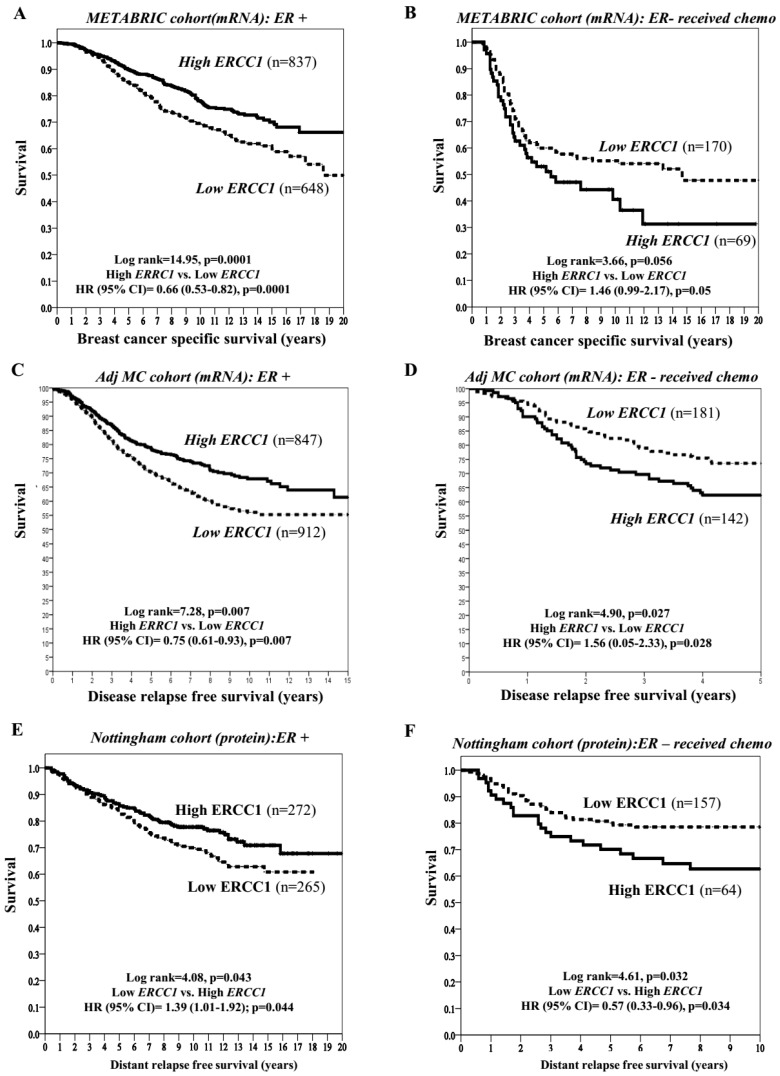
*ERCC1*, adjuvant chemotherapy and survival. (**A**) Kaplan Meier curves showing BCSS (Breast cancer specific survival) based on *ERCC1* mRNA expression in ER+ METABRIC cohort. (**B**) Kaplan Meier curves showing BCSS (Breast cancer specific survival) based on *ERCC1* mRNA expression in ER− METABRIC cohort. (**C**) Kaplan Meier curves showing disease specific survival based on *ERCC1* mRNA expression in ER+ Multicentre Adjuvant (Adj MC) cohort. (**D**) Kaplan Meier curves showing disease specific survival based on *ERCC1* mRNA expression in ER− Adj MC cohort. (**E**) Kaplan Meier curves showing disease specific survival based on ERCC1 protein level in ER+ Adj MC cohort. (**F**) Kaplan Meier curves showing disease specific survival based on ERCC1 protein level in ER− Adj MC cohort.

In the ER+ METABRIC whole cohort, low *ERCC1* transcript was associated with higher risk of death (*p* = 0.0001) (Figure 1A). In patients who received endocrine therapy, similarly, low *ERCC1* was associated with higher risk of death (*p* = 0.0001) (Figure S2A). In addition, in the ER+ METABRIC cohort, we tested 188 DNA repair genes in a multivariate Cox proportional hazards model with backward stepwise exclusion and identified *ERCC1* (among seven other genes) as an independent predictor for overall survival (OS) (Table S10). As chromosomal instability is a marker of chemo-sensitivity, we evaluated *ERCC1* in ER− METABRIC patients who received chemotherapy. Low *ERCC1* mRNA expression was associated with a decreased risk of death from BC (*p* = 0.05) (Figure 1B). In a multivariate Cox regression analysis after controlling for confounders (such as endocrine therapy, chemotherapy and other validated prognostic factors (ER, PR, HER2, grade, stage, tumour size, TP53 mutation status, PAM50 molecular subtype and IntClust subclasses)), we confirmed that *ERCC1* transcript was an independent prognostic factor for OS (*p* = 0.039) and the interaction between *ERCC1* and chemotherapy was also statistically significant (*p* = 0.020) (Table 2). In ER− tumours, that received no chemotherapy, ERCC1 did not influence survival (Figure S2B). 

We then validated in the Multicentre (MC)-Adjuvant cohort of 4640 patients. By using mean as cut-off, high and low *ERCC1* were observed in 49% (1460/2261) and 51% (14602379) of cases, respectively. ER and HER2 status were available for 3826 and 1727 cases; respectively. About 59% (2268/3826), 41% (1558/3826) and 26% (446/1727) of cases were ER+, ER− and HER2+, respectively (Tables S3 and S4). Similar to the METABRIC data, in ER+ tumours, low levels of *ERCC1* was associated with an increased distant relapse DRR compared to high levels of *ERCC1* [*p* = 0.007) (Figure 1C). However, in ER− tumours that received adjuvant chemotherapy, low *ERCC1* was associated with a reduced DRR compared to high *ERCC1* (*p* = 0.001) (Figure 1D). Multivariable Cox regression models confirmed that the low *ERCC1* transcript is a poor prognostic factor for DRR after controlling with Adjuvant! Online (AOL) (*p* = 0.047) and 72-proliferation-gene-signatures (*p* < 0.0001).

### 2.3. ERCC1 Protein, Clinicopathological Features and Outcomes

Using median as the cut off (*H*-score ≥ 130), we observed ERCC1 nuclear protein expression in 439/991 (44.3%) of breast tumours, and 55.7% (552/991) were negative for ERCC1 expression. As shown in (Table S11), low nuclear ERCC1 level was significantly associated with aggressive phenotypes, including high grade, no special histological type (NST), ER−, basal-like phenotype and triple negative tumours, as well as loss of other DNA repair biomarkers (all adjusted *p* ≤ 0.01). In ER+ tumours, low ERCC1 protein was linked to poor disease relapse free survival (*p* = 0.044) (Figure 1E). On the other hand, in ER− tumours that received chemotherapy, low ERCC1 protein was linked to improved disease relapse free survival (*p* = 0.034)) (Figure 1F). Multivariable Cox regression analysis controlling for chemotherapy, endocrine therapy and other validated prognostic factors (stage, grade, size, ER, PR, HER2 and BCl2), showed that ERCC1 protein expression was an independent prognostic factor for OS (*p* = 0.035) and that the interaction between ERCC1 protein expression and adjuvant chemotherapy was statistically significant (*p* = 0.022) (Table S12).

### 2.4. ERCC1 and Pathological Complete Response (pCR) to Neoadjuvant Chemotherapy

Neoadjuvant chemotherapy (pre-operative) is an established approach in locally advanced breast cancers (LABC). Although current evidence suggests that patients who achieve pCR have a better long-term clinical outcome [27,28], the development of a predictive biomarker of pCR remains a high priority. We therefore evaluated *ERCC1* transcripts in a multiple centre cohort of 2345 LABC patients who received neoadjuvant anthracycline based combination (Neo-Adj) AC-chemotherapy (CT) + or − T with or without Herceptin (+ or – H), including multiple clinical trials sub cohorts (MC-Neo-Adjuvant cohort). The majority of patients (60%; 1413/2345) had received Neo-Adj AC-CT+T (taxane) whereas 29% (689/2345) and 10% (243/2345) of patients had received Neo-Adj AC-CT alone and AC-CT+T+H; respectively. About 52% of cases were ER− (1163/2256) whereas 48% (1093/2345) and 24% (518/2163) were ER+ and HER2+, respectively. Low and high ERCC1 transcript expressions were observed in 48% (1133/2345) and 52% (1212/2345) of cases, respectively. Out of the 2345 patients, 596 (25%) patients had achieved pCR. Low *ERCC1* transcript expression was associated with an increased proportion of patients achieving pCR (333 (29%) of 1133 patients) compared with high *ERCC1* transcript expression (263 (22%) of 1212 patients; OR (95% CI): 1.50 (1.25–1.81, *p* < 0.0001).

**Figure 2:**
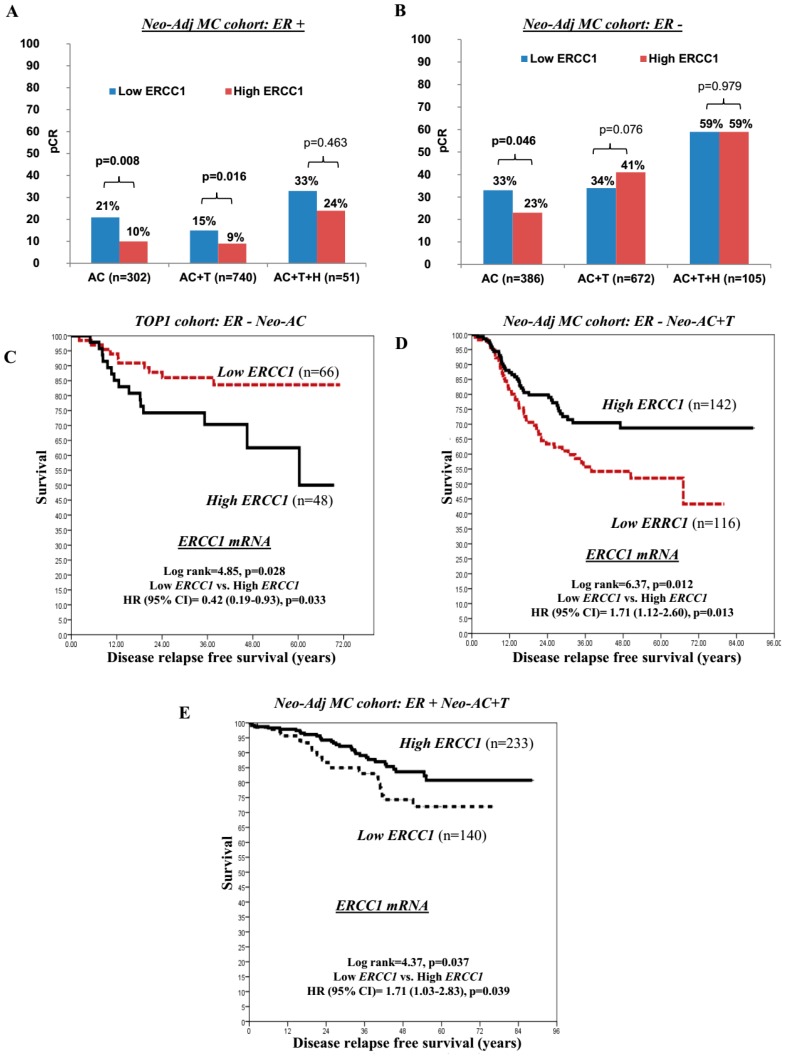
ERCC1 and neoadjuvant chemotherapy. (**A**) Pathological complete response (pCR) based on *ERCC1* mRNA expression in ER+ tumours (neoadjuvant anthracycline based (Neo-Adj) MC cohort) that received neoadjuvant AC or AC+T or AC+T+H chemotherapy. (**B**) Pathological complete response (pCR) based on *ERCC1* mRNA expression in ER− tumours (Neo-Adj MC cohort) who received neoadjuvant AC or AC+T or AC+T+H chemotherapy. (**C**) Disease free survival based on *ERCC1* mRNA expression in TOP1 cohort patients who received neoadjuvant AC chemotherapy. (**D**) Disease free survival based in ER− Neo-Adj MC cohort who received neoadjuvant AC+T chemotherapy. (**E**) Disease free survival based in ER+ Neo-Adj MC cohort who received neoadjuvant AC+T chemotherapy.

In ER+ patients, low *ERCC1* transcript expression was also associated with a higher proportion of patients achieving pCR (80 (18%) of 442 patients) compared with high *ERCC1* transcript expression (64 (10%) of 651patients; OR (95% CI): 2.03 (1.42–2.89), *p* < 0.0001) especially in ER+ patients who received either Neo-Adj AC-CT alone (21% (29/136) versus 10% (17/166); OR (95% CI): 2.38 (1.24–4.55), *p* = 0.008) or Neo-Adj ACT-CT+T (15% (41/276) versus 9% (42/464); OR (95% CI): 1.75 (1.11–2.77), *p* = 0.016) (Figure 2A).

In ER− patients who received Neo-Adj AC-CT alone, low *ERCC1* transcript expression was also associated with a higher proportion of patients achieving pCR (74 (33%) of 227 patients) compared with high *ERCC1* transcript expression (37 (23%) of 159 patients (OR (95% CI): 1.59 (1.01–2.53), *p* = 0.046) (Figure 2B). We validated this observation in the TOP1 trial cohort of ER-negative tumours where patients received anthracycline (epirubicin) monotherapy only. Low *ERCC1* transcript expression was associated with an increased proportion of patients achieving pCR (12 (21.4%) of 56 patients) compared with high *ERCC*1 transcript expression (4 (6.9%) of 58 patients; OR (95% CI): 3.683 (1.11–12.20), *p* = 0.026). Moreover, in the TOP1 cohort, low *ERCC1* transcript expression was associated with 58% lower relapse risk compared to high ERCC1, (HR (95% CI): 0.42 (0.19-0.93); *p* = 0.033) (Figure 2C). For additional validation at protein level, we investigated the effect of ERCC1 protein on pCR in a series of 120 LABC patients who received Neo-Adj AC-CT alone. 19/120 (16%) patients achieved pCR in this cohort. Low ERCC1 protein expression was associated with an increased proportion of patients achieving a pCR (16 (26%) of 62 patients) compared with high ERCC1 protein expression (3 (5%) of 57 patients; OR (95% CI): 6.25 (1.89–22.73, *p* = 0.002).

On the other hand, in ER− patients who received Neo-Adj ACT+T, high *ERCC1* transcript expression was associated with a higher proportion of patients achieving a pCR (142 (41%) of 349 patients) compared with low *ERCC1* transcript expression (110 (34%) of 323 patients; OR (95% CI): 1.33 (0.97–1.82), *p* = 0.076) (Figure 2B). In addition, in ER− patients who received pre-operative Neo-Adj ACT+T, low *ERCC1* had higher relapse risk compared to high *ERCC1* (HR (95% CI): 1.71 (1.12–2.60); *p* = 0.013) (Figure 2D). Similarly, in ER+ patients also who received Neo-Adj ACT+T, low *ERCC1* had higher relapse risk compared to high *ERCC1* (hazard ratio (HR) (95% CI) = 1.71 (1.03–2.83), *p* = 0.039) (Figure 2E).

Taken together, the data provides compelling evidence that ERCC1 has prognostic significance in ER+ BCs and predict response to chemotherapy in ER− BCs. 

## 3. Discussion

Although the efficacy of DNA damaging chemotherapy (such as anthracyclines) is influenced by impaired DNA repair capacity, evolving evidence also suggests that mitotic spindle poisons (such as taxanes) are more effective in low grade chromosomally stable tumours. Therefore the development of robust DNA repair based biomarkers is highly desirable. ERCC1 is non-catalytic but partners with XPF endonuclease to form the ERCC1–XPF heterodimer which processes abnormal DNA repair intermediates generated during NER, double strand breaks (DSB) repair and Interstrand Cross Link (ICL) repair [29]. Given the key role for ERCC1 in genomic integrity, we hypothesized a role for ERCC1 in breast cancer pathogenesis and response to therapy. In the current study we show that *ERCC1* transcript expression was independently associated with OS in the METABRIC cohort. In ER+ tumours, low *ERCC1* transcript or protein level was associated with increased distant relapse risk (DRR). In ER− tumours, low *ERCC1* transcript or protein level was linked to decreased DRR, especially in patients who received anthracycline chemotherapy. In LABC patients who received neoadjuvant anthracycline, low *ERCC1* transcript was associated with higher pCR (pathological complete response) and decreased DRR. However, in patients with ER−tumours who received additional neoadjuvant taxane, high *ERCC1* transcript was associated with a higher pCR and decreased DRR. High *ERCC1* transcript was also linked to decreased DRR in ER+ LABC that received additional neoadjuvant taxane. Taken together, the data presented here provides comprehensive clinical evidence that ERCC1 is a predictor of anthracycline resistance and taxane sensitivity in breast cancers. ERCC1 based stratification could be an attractive strategy in breast cancers. 

Studies exploring biomarkers of response to anthracycline therapy have been limited in breast cancers. Previous smaller studies suggest that Ki67, HER1-3 expression, TOP2A and HER-2 are potential markers of anthracycline benefit [30]. Ch17CEP, a CIN marker, was also previously shown to predict benefit from anthracycline adjuvant chemotherapy in a prospective clinical trial [8]. ERCC1 is a critical factor for CIN. To the best of our knowledge, the data shown here represents the first comprehensive evidence that ERCC1 status influences potential benefitting from anthracycline chemotherapy. The role of ERCC1 in breast cancer pathogenesis is emerging. ERCC1 polymorphism may be associated with increased breast cancer risk [31]. A previous, small study suggested that ERCC1 protein levels may be low in triple negative breast cancers (TNBCs) [32,33]. In a study of fifty two TNBCs, ERCC1 positivity was associated with shorter progression free survival and poor response to neoadjuvant chemotherapy [32]. In addition, *ERCC1* genetic polymorphism also appeared to associate with pCR in patients receiving neoadjuvant anthracycline chemotherapy [34]. Gay-Beillile et al. recently also demonstrated that *ERCC1* expression is induced in tumours that receive anthracycline based neoadjuvant chemotherapy [35]. Previous clinical studies have evaluated the predictive significance of ERCC1 for response to platinum chemotherapy, in various solid tumours (lung, colorectal, head and neck, gastric and bladder cancers [36,37,38,39]. However, a major limitation has been the use of relatively non-specific ERCC1 antibodies for immunohistochemistry in previous studies, including in a large lung cancer clinical trial [40]. In the current study we utilised a recently generated and highly specific mouse monoclonal antibody (clone 4F9) [41], which further strengthens our clinical data. Our study not only concurs with previous studies showing a link between ERCC1 overexpression and chemoresistance but also provides additional insight suggesting that ERCC1 may also be involved in the emergence of aggressive breast cancer phenotypes. However, a limitation to our study is that it is predominantly retrospective. Prospective studies would be required to confirm our findings.

Currently there is no established predictive biomarker of response to taxane therapy. Previous studies suggest that HER2, Ki67, class III β tubulin expression may influence taxane response [10]. A novel observation in the current study is that ERCC1 was also shown to influence whether taxane chemotherapy was beneficial. Our data concurs with previous evidence demonstrating taxane benefit in low grade, chromosomally stable tumours [9,10]. However, further prospective studies would be required to confirm our initial findings.

Taken together, the data would support further development of ERCC1 as a biomarker of response to chemotherapy in breast cancer.

## 4. Patients and Methods

### 4.1. Study Design and Cohorts

**Figure 3:**
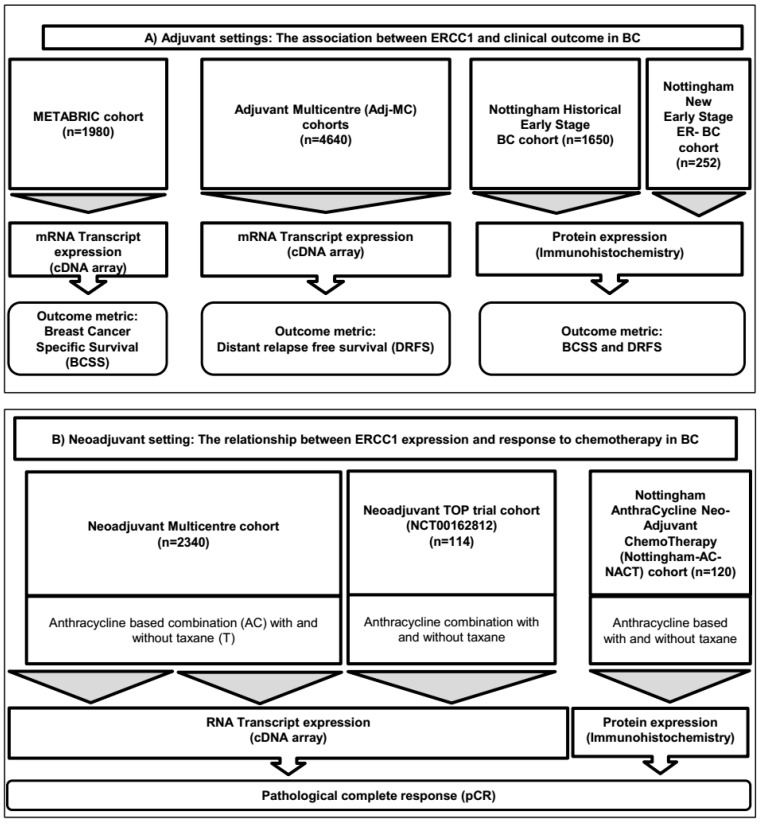
Consort diagram summarizing patient cohorts investigated in the current study.

Study design, the patient cohorts which included 11,096 BCs and their demographics are summarized in the consort flow diagram (Figure 3), also in Supplementary Methods and Tables.

### 4.2. Outcome Measurements and Patient Cohorts:

#### 4.2.1. *ERCC1* Transcript Expression Analysis

I The association of 188 DNA repair genes and prognosis (overall survival; OS) analysis:

Cohort (1): METABRIC cohort (Molecular Taxonomy of BC International Consortium)

Patient demographics are summarized in Supplementary Table S1. We investigated the association of 188 DNA repair genes (Table S2) with OS in the METABRIC cohort (METABRIC *n* = 1980; median follow-up time in years (MFUT) (inter-quantile range (IQR): 9.1 (5.2–12.9)). Univariate Cox regression analysis was used in SPSS (Version 20, Chicago, IL, USA) and the Benjamini and Hochberg False Discovery Rate calculation (BH FDR) was applied to account for multiple comparisons. After definition of factors that were associated with OS after BH FDR correction, multivariate Cox proportional hazards models (with backward stepwise exclusion of these factors, using a criterion of *p* < 0.05 for retention of factors in the model) were used to identify factors that were independently associated with OS. The statistical significance of the model was assessed based on the likelihood ratio test. The proportional hazards assumption was tested using both standard log–log plots and by generating Kaplan–Meier survival estimate curves, and observing that the curves did not intersect with each other. Hazard ratios (HRs) for death risk and 95% confidence intervals were calculated from the Cox proportional hazards analysis. 

II The association of distant relapse risk (DRR)) and *ERCC1* transcript after receiving systemic adjuvant therapy (Adj-T):

Cohort (2): Multicentre (MC)-Adjuvant cohort (*n* = 4640)

The association between *ERCC1 mRNA* expression and DRR and its relationship with the received systemic Adj-T were tested in 4640 patients with early stage BC, retrieved from 21 gene expression databases (see Supplementary Tables S3 and S4). ERCC1 gene expression data of each database were converted to a common scale (median equal to 0 and standard deviation equal to 1) in order to merge all of the study data that used the same platform and to create combined cohorts. Then the data was median centred for each gene, whereby the median of each gene was 0. Databases using the same platform were merged and the median expression was calculated. The median expression of *ERCC1* transcription for each platform was calculated and values equal to or higher than the median coded as +1 (overexpression). Values of less than the median were coded 0 or low ERCC1. Distal relapse free survival follow up data were available for 3171 patients with 967 events (MFUT (IQR): 5.5 (3.0–8.7)). The systemic Adj-T information was available for 2276 patients: 45% of patients were naïve to systemic Adj-T, whereas 49% had received Adj-endocrine therapy and 27% had received chemotherapy. Herceptin had been offered to 156 (7%) of HER2 + patients.

III The association between *ERCC1* and pathological complete response rate (pCR) analysis after receiving neoadjuvant anthracycline based combination chemotherapy (Neo-Adj-ACT)

Cohort (3): Multicentre (MC) Neo-Adjuvant cohort (*n* = 2340)

Demographics summarized in Supplementary Table S5. The association between *ERCC1* mRNA expression and pCR was evaluated in 2340 patients retrieved from 15 gene expression databases that received pre-operative anthracycline based combination (AC) with (+) or without (−) taxane (T). Out of 2345 patients, 689 patients (29%) has received Neo-Adjuvant anthracycline based combination chemotherapy (Neo-Adj-ACT) alone; 1413 patients (60%) received Neo-Adj-ACT with Taxane (ACT + T) and 243 patients received Neo-Adj-ACT + T + Herceptin (ACT + T + H).

Cohort (4): Neoadjuvant TOP trial cohort (NCT00162812), in which patients with oestrogen receptor (ER)-negative tumours were treated with anthracycline (epirubicin) monotherapy. Demographics summarized in Supplementary Table S6.

#### 4.2.2. Protein Expression Association Analysis

Immunohistochemical evaluation of ERCC1 protein expression was performed in three cohorts of patients who treated at a single centre (Nottingham University Hospital (NUH)). We utilised a recently characterised highly specific anti-ERCC1 mouse monoclonal antibody (clone 4F9, Dako Ltd., Cheshire, UK) [18]. We confirmed the specificity of clone 4F9, using Western Blots in breast cancer (SKBR3, T47D and MDA-MB-231) and ovarian cancer (A2780 and A2780cis) cell lines (Supplementary Figure S1A). Tissue culture and Western blot analyses is described in Supplementary Methods.

### 4.3. Adjuvant Setting

Cohort (5): NUH- early stage breast cancer (NUH-ESBC): The study was performed in a well characterised consecutive series of 1650 patients with primary invasive breast carcinomas who were diagnosed between 1986 and 1999 and entered into the Nottingham Tenovus Primary Breast Carcinoma series [42] (Nottingham historical early stage cohort (NUH-ESBC); MFUT (IQR): 13.4 (10.3–16.42)). Demographics are summarized in Supplementary Table S7. Supplementary Methods provide a detailed description on Tissue Microarrays (TMAs) and immunohistochemistry (IHC) evaluations (Table S8). The association between the ERCC1 protein expression with clinicopathological parameters and DRR were analysed in this cohort.

Cohort (6): (Nottingham ER-negative series). A series of 252 ER negative invasive breast carcinomas diagnosed and managed at the Nottingham University Hospitals (NUH) Trust between 1999 and 2007. All patients were primarily treated with surgery, followed by adjuvant radiotherapy and anthracycline based combination chemotherapy [43]. Demographics are shown in Supplementary Table S9.

### 4.4. Neo-Adjuvant Setting

Cohort (7): Nottingham anthracycline based neo-adjuvant chemotherapy cohort (Nottingham AC-NACT; *n* = 120) consisting of pre-chemotherapy core biopsies from 120 female patients with locally-advanced primary BC treated with neo-adjuvant (Neo-Adj) anthracycline-based combination chemotherapy (AC-CT) (Neo-Adj-AC-CT) treated at NUH between 1996 and 2012 [42].

All patients completed written informed consented, as per hospital standard of care, for excess tumour tissue to be used in research. The study was approved by the Institutional Review Board or Independent Ethics Committee and the Hospital Research and Innovations Department at all participating sites. Tumour Marker Prognostic Studies (REMARK) criteria, as recommended by McShane et al. [44] were followed throughout this study.

### 4.5. Power Analysis

A retrospective power analysis was conducted to determine the confidence in the calculated hazard ratio and associated *p* value for 10 year survival and to ascertain how applicable the result would be to a global population. Power of study was determined using PASS (NCSS, version 13, USA).

### 4.6. Statistical Analysis

Statistical analyses were performed using STATISTICA (Stat Soft Ltd., Tulsa, OK, USA) and SPSS (version 17, Chicago, IL, USA) by the authors who were blinded to the clinical data. Where appropriate, Pearson’s chi-squared, student’s t-test and ANOVA tests were used. Positivity for ERCC1 protein both pre- and post-chemotherapy was calculated and compared using McNemar’s test. Cumulative survival probabilities and 10-year BCSS and DFS were estimated using the univariate Cox proportional hazards models and the Kaplan–Meier plot method where appropriate, and differences between survival rates were tested for significance using the log-rank test. Multivariable analysis for survival was performed using the Cox proportional hazard model. The proportional hazards assumption was tested using standard log–log plots. Hazard ratios (HR) and 95% confidence intervals (95% CI) were estimated for each variable. All tests were two-sided with a 95% CI, and a *p* value < 0.05 was considered to be indicative of statistical significance. The interaction between ERCC1 and chemotherapy was tested in the Cox proportional hazard model. For multiple comparisons, *p* values were adjusted according to Benjamini–Hochberg method [45]. Tumor Marker Prognostic Studies (REMARK) criteria, recommended by McShane et al. [46], were followed throughout this study. Ethical approval was obtained from the Nottingham Research Ethics Committee (C202313).

## 5. Conclusions

ERCC1 is non-catalytic but partners with XPF endonuclease to form the ERCC1–XPF heterodimer which processes abnormal DNA repair intermediates generated during NER, DSB repair and ICL repair. We provide the first comprehensive clinical evidence that ERCC1 is a key predictor of chemotherapy response in patients with breast cancer who receive adjuvant or neoadjuvant chemotherapy. Importantly, the clinical study suggests that ERCC1 based stratification is feasible in BC patients who receive anthracycline and/or taxane chemotherapy.

## Figures and Tables

**Table 1:** Clinicopathological significance of *ERCC1* mRNA expression in breast cancers.

	*ERCC1* mRNA Expression		*p*-Value	* *p*-Value(Adjusted)
	Low	High		
**(A) Pathological Parameters**				
Tumour Size				
≤1cm	43 (4.5%)	43 (4.4%)	0.481	5.2910
>1–2cm	247 (25.7%)	279 (28.8%)		
>2–4cm	620 (64.5%)	601 (62.0%)		
>4cm	51 (5.3%)	46 (4.7%)		
Tumour Grade				
1	35 (3.7%)	130 (14.1%)	4.4 × 10^−37^	<0.00001
2	305 (32.0%)	460 (49.8%)		
3	612 (64.3%)	334 (36.1%)		
Lymph Node Group				
Negative	486 (49.8%)	528 (54.2%)	0.051	0.0623
Positive	490 (50.2%)	446 (45.8%)		
Histological Types				
IDC-NST	837 (85.8%)	704 (72.3%)	1.33 × 10^−15^	<0.00001
Medullary Carcinoma	20 (2.0%)	12 (1.2%)		
Invasive special type	104 (10.7%)	247 (25.4%)		
Invasive others	15 (1.5%)	11 (1.1%)		
Ki67 Expression				
Negative	375 (38.4%)	600 (61.6%)	1.37 × 10^−24^	<0.00001
Positive	601 (61.6%)	374 (38.4%)		
P53 Mutation				
Wild type	325 (82.9%)	383 (92.3%)	4.9 × 10^−5^	<0.00001
Mutant	67 (17.1%)	32 (7.7%)		
ER Expression				
Negative	332 (34.0%)	126 (12.9%)	4.8 × 10^−28^	<0.00001
Positive	644 (66.0%)	848 (87.1%)		
PAM 50 Luminal A				
Negative	770 (78.9%)	466 (48.1%)	4.45 × 10^−45^	<0.00001
Positive	206 (21.1%)	502 (51.9%)		
PAM 50 Luminal B				
Negative	684 (70.1%)	775 (80.1%)	3.68 × 10^−7^	<0.00001
Positive	292 (29.9%)	193 (19.9%)		
PAM 50 Her2				
Negative	799 (81.9%)	909 (93.9%)	4.39 × 10^−16^	<0.00001
Positive	177 (18.1%)	59 (6.1%)		
PAM 50 Basal				
Negative	751 (76.9%)	871 (90.0%)	1.089 × 10^−14^	<0.00001
Positive	225 (23.1%)	97 (10.0%)		
Integrative Molecular Clusters				
Int Clust 1	101 (10.3%)	35 (3.6%)	1.163 × 10^−60^	<0.00001
Int Clust 2	41 (4.2%)	30 (3.1%)		
Int Clust 3	78 (8.0%)	210 (21.6%)		
Int Clust 4	144 (14.8%)	187 (19.2%)		
Int Clust 5	139 (14.2%)	46 (4.7%)		
Int Clust 6	52 (5.3%)	33 (3.4%)		
Int Clust 7	74 (7.6%)	112 (11.5%)		
Int Clust 8	77 (7.9%)	221 (22.7%)		
Int Clust 9	108 (11.1%)	38 (3.9%)		
Int Clust 10	162 (16.6%)	62 (6.4%)		
Genufu Sub-Types				
ER−/Her-2−	104 (21.4%)	44 (8.8%)	7.43 × 10^−37^	<0.00001
ER+/Her-2– (high proliferation)	212 (43.7%)	148 (29.7%)		
ER+/Her-2– (low proliferation)	85 (17.5%)	281 (56.3%)		
Her-2 +	84 (17.3%)	26 (5.2%)		

* *p* values were adjusted according to Benjamini-Hochberg method.

**Table 2:** Multivariate Cox regression analysis for overall survival (OS) at 20 years in the METABRIC cohort.

Variables	HR	95.0% CI		*p* Value
		Lower	Upper	
*ERCC1* mRNA expression (+)	1.43	1.02	2.01	0.039 *
ER (+)	0.75	0.38	1.49	0.411
PR (+)	0.91	0.63	1.32	0.624
HER2 overexpression	0.82	0.36	1.85	0.63
TP53 mutation	1.81	1.24	2.63	0.002 *
Tumour Size (continuous)	1.01	1.01	1.02	0.001*
Lymph node (LN) stage				1.2 × 10^−6^ *
Negative	1			
1–3 positive LNs	1.87	1.25	2.79	
> 3 positive LNs	3.33	2.12	5.23	
Histological grade				0.563
Low	1			
Intermediate	0.98	0.46	2.09	
High	1.2	0.55	2.62	
PAM-50 subtypes				0.049 *
PAM-50-LUM A	1	0.97	2.16	
PAM-50-LUM B	1.44	0.43	2.16	
PAM-50-LUM HER2	0.96	0.5	3.04	
PAM-50-LUM Basal	1.24	1.17	4.56	
PAM-50-Normal like	2.31			
IntClust Members				0.189
IntClust 1	1			
IntClust 2	1.28	0.59	2.8	
IntClust 3	0.56	0.25	1.24	
IntClust 4	0.8	0.4	1.59	
IntClust 5	2.47	0.94	6.53	
IntClust 6	1.15	0.5	2.64	
IntClust 7	1.08	0.49	2.38	
IntClust 8	1.03	0.52	2.05	
IntClust 9	1.31	0.66	2.58	
IntClust 10	0.81	0.38	1.76	
Hormone therapy	0.64	0.43	0.96	0.031 *
Chemotherapy	0.93	0.62	1.41	0.741
Interaction term	2.86	1.1	7.42	0.09
Hormone therapy * ER (IHC)				
Interaction term	2.11	1.23	3.95	0.020 *
Chemotherapy * ERCC1				

* significant *p* values.

## Supplementary Materials

The following are available online at https://www.mdpi.com/2072-6694/11/8/1149/s1, Table S1: Clinicopathological characteristics in the METABRIC cohort, Table S2: List of DNA repair genes tested in the METABRIC cohort, Table S3: Cohort ID and gene expression platform Multicentre (MC)-Adjuvant cohort (n=4640), Table S4: Demographics of Multicentre (MC)-Adjuvant cohort (n=4640), Table S5: Multicentre (MC) Neo-Adjuvant cohort (n= 2345), Table S6: Demographics in TOP trial cohort, Table S7: Clinicopathological characteristics of Nottingham historical early, TableS8: Table of antibodies and optimisation conditions used to immunohistochemically profile the Nottingham University Hospitals based cohorts. Detailed below are: Antigens, primary antibodies, clone, source, optimal dilution and scoring system, used for each immunohistochemical marker stage cohort (NUH-ESBC), Table S9: Clinicopathological characteristics of ER- cohort, Table S10: Multivariate backward step-wise Cox analysis for DNA repair genes, associated with Overall Survival (OS) in ER+ METABRIC cohort, Table S11: Clinicopathological significance of ERCC1 protein expression in breast cancers, Table S12: Multivariate Cox regression analysis for breast cancer specific survival (BCSS) at 20 years follow up in Nottingham series including interaction terms.
