# Supplementary material for: ERCC1 Is a Predictor of Anthracycline Resistance and Taxane Sensitivity in Early Stage or Locally Advanced Breast Cancers
Source: Cancers (Basel). 2019 Aug 10;11(8):1149. doi: 10.3390/cancers11081149 (PMC6721618; doi:10.3390/cancers11081149)
Supplement: Supplementary file 1 [file cancers-11-01149-s001.pdf]

Supplementary Methods

Tissue Microarrays (TMAs) and immunohistochemistry (IHC) evaluations: Tumours were arrayed in tissue microarrays (TMAs) constructed with 2 replicate 0.6mm cores from the centre and periphery of the tumours. The TMAs were immunohistochemically profiled for ERCC1 and other biological antibodies (**Supplementary Table S4**) as previously described. Immunohistochemical staining was performed using the Thermo Scientific Shandon Sequenza chamber system (REF: 72110017), in combination with the Novolink Max Polymer Detection System (RE7280-K: 1250 tests), and the Leica Bond Primary Antibody Diluent (AR9352), each used according to the manufacturer's instructions (Leica Microsystems). The tissue slides were deparaffinised with xylene and then rehydrated through five decreasing concentrations of alcohol (100%, 90%, 70%, 50% and 30%) for two minutes each. Pre-treatment antigen retrieval was performed on the TMA sections using sodium citrate buffer (pH 6.0) and heated for 20 minutes at 95°C in a microwave (Whirpool JT359 Jet Chef 1000W). A set of slides were incubated for 18 hours with the primary anti-ERCC1 mouse monoclonal antibody [clone 4F9 (catalogue number: M3648), Dako Ltd, UK], at a dilution of 1:50 incubated for 60 minutes. Negative and positive (by omission of the primary antibody and IgG-matched serum) controls were included in each run. The negative control ensured that all the staining was produced from the specific interaction between antibody and antigen.

Whole field inspection of the core was scored and intensities of nuclear staining were grouped as follows: 0 = no staining, 1 = weak staining, 2 = moderate staining, 3 = strong staining. The percentage of each category was estimated (0-100%). Histochemical score (H-score) (range 0-300) was calculated by multiplying intensity of staining and percentage staining. For breast cancers, a median H score of ≥ 130 was taken as the cut-off for high ERCC1 nuclear expression. For ovarian cancers, a median H score of ≥ 130 was taken as the cut-off for high ERCC1 nuclear expression. Not all cores within the TMA were suitable for IHC analysis as some cores were missing or lacked tumour (<15% tumour).

Expression of HER2, ER and PR was re-assessed according to the American Society of Clinical Oncology/College of American Pathologists (ASCO/CAP) guidelines . To validate the use of TMAs for immuno-phenotyping, full-face sections of 40 cases were stained and the protein expression levels were compared. The concordance between TMAs and full-face sections was excellent using Cohen's kappa statistical test for categorical variables (kappa=0-8). Positive and negative (omission of the primary antibody and IgG matched serum) controls were included in each run.

Determination of the cut-offs: The median in each cohort was used as cut-off between low and high expression gene/protein expression

The clinicopathological and biomarkers associations: The clinicopathological and molecular characteristics of *ERCC1* transcript were determined in the METABRIC and MCC cohorts. The associations between ERCC1 protein expression and clinicopathological parameters, as well as prognostic biomarkers, were analysed in the Nottingham-HES-BC cohort. The clinicopathological parameters including mainly: tumour size, lymph node stage, histological grade, lympho-vascular invasion, histological tumour types, genomic grade index (GGI), TP53 mutation, intrinsic molecular subclasses, PAM50, HER2 amplification/overexpression, hormone receptors, Ki67 and mitotic index.

Breast cancer specific survival (BCSS): *The ERCC1* transcript expression association with BCSS was explored in METABRIC and LN-negative untreated Desmedt et al cohorts. The association between ERCC1 protein expression and BCSS was analysed in Nottingham-HES-BC cohort. Survival data were maintained on a prospective basis. Breast cancer specific survival (BCSS) was defined as the number of months from diagnosis to the occurrence of breast cancer related death. Survival was censored if the patient was still alive, lost to follow-up, or died from other causes.

Distant relapse free survival (DRFS): To test *ERCC1* transcript expression as a biomarker for BC outcome, the association with DRFS has been analysed in MCC and therapy naïve Schmidt *et al* and Desmedt *et al* cohorts (n=349). Furthermore, to test ERCC1 transcript expression as a predictive biomarker for outcome after neoadjuvant combination cytotoxic chemotherapy, we investigated its association with

DRFS in the MD Anderson-Neo-ACT cohort (n=509) and TOP1 clinical trial. DRFS was defined as the number of months from diagnosis to DM relapse. The relationship between ERCC1 protein expression, chemotherapy and DRFS was tested in Nottingham adjuvant cohorts.

Pathological response rate (pCR): To assess ERCC1 transcript expression as a predictive biomarker for response to combination cytotoxic chemotherapy, we analysed the association with pCR in MD Anderson-Neo-ACT, phase 2 Neo-ACT clinical trial cohorts and ER negative TOP1 clinical trial. ERCC1 protein and p CR was evaluated in Nottingham AC-Neo-ACT cohort .

Power analysis: A retrospective power analysis was conducted to determine the confidence in the calculated hazard ratio and associated p value for 10 year survival and to ascertain how applicable the result would be to a global population. Power of study was determined using PASS (NCSS, version 13, USA).

Statistical analysis: Statistical analyses were performed using STATISTICA (Stat Soft Ltd, Tulsa, USA) and SPSS (version 17, Chicago, USA) by the authors who were blinded to the clinical data. Where appropriate, Pearson's chi-squared; student's t-test and ANOVA tests were used. Positivity for ERCC1 protein both pre- and postchemotherapy was calculated and compared using McNemar's test. Cumulative survival probabilities and 10-year BCSS and DFS were estimated using the univariate Cox proportional hazards models and the Kaplan-Meier plot method where appropriate, and differences between survival rates were tested for significance using the log-rank test. Multivariable analysis for survival was performed using the Cox proportional hazard model. The proportional hazards assumption was tested using standard log-log plots. Hazard ratios (HR) and 95% confidence intervals (95% CI) were estimated for each variable. All tests were two-sided with a 95% CI and a p value <0-05 was considered to be indicative of statistical significance. The interaction between ERCC1 and chemotherapy was tested in Cox proportional hazard model. For multiple comparisons, p values were adjusted according to Benjamini-Hochberg method (16). Tumor Marker Prognostic Studies (REMARK) criteria, recommended by McShane et al {McShane, 2005 #90}, were followed throughout this study. Ethical approval was obtained from the Nottingham Research Ethics Committee (C202313).

Tissue culture and Western blots: A panel of breast cancer cell lines **[**T47D, SKBR3 and MDA-MB-23] and ovarian cancer cell lines [A2780, A2780cis] were profiled for ERCC1 expression. All cell lines were purchased from ATCC and authenticated by ATCC. Cells were grown in RPMI (MCF-7, A2780, A2780 Cis, PE01,PE04, OVCAR3,OVCAR4 & SKOV3), MEM (MDA-MB-231), Mccoy 5A(SKBR3), DMEM high glucose (T47D) or DMEM-F12 (MDA-MB-468 & MCF-10 A) medium supplemented with 10% foetal bovine serum or 15% horse raddish serum (MCF-10A) and 1% penicillin/streptomycina. Western blotting for ERCC1 was performed as described before (REFERENCES). Primary anti-ERCC1 antibody [clone 4F9 (catalogue number: M3648), Dako Ltd, UK] was incubated over night at room temperature at a dilution of 1:1500. Primary anti-β actin antibody (1:10000 dilution [Abcam]) was used as a loading control. Infrared dye-labelled secondary antibodies (Li-Cor) [IRDye 800CW Mouse Anti-Rabbit IgG and IRDye 680CW Rabbit Anti-Mouse IgG] were incubated at a dilution of 1:10000 for 1 hour. Membranes were scanned with a Li-Cor Odyssey machine (700 and 800nm) to determine protein expression.

Supplementary Table S1: Clinicopathological characteristics in the METABRIC cohort

Variables	N (%)
**Age at diagnosis** [Median (range)]	61.8 (21.93-96.29)
**Tumour size** [Median (range)]	23 (1, 182)
**NPI** [Median (95% CI)]	4.04 (3.99-4.09)
**Survival** [Median (Months, 95% Cl)]	149 (141-159)
**Lymph nodes status**	
0	1012
1	336
2	170
3	112
>3	316
**ER status**	
Positive	1485
Negative	437
**PAM50 subtype**	
Basal	322
HER2	238
Luminal A	714
Luminal B	484
Normal	188

Not classified	6
**Adjuvant systemic therapy (AT)**	
No AT	290
Hormone therapy (HT)	1014
Chemotherapy	226
Hormone + chemotherapy	192

Supplementary Table S2: List of DNA repair genes tested in the METABRIC cohort.

**Genes**	**Symbol**
ALKBH2 (ABH2)	ALKBH2
ALKBH3 (DEPC1)	ALKBH3
APEX1 (APE1)	APEX1
APEX2	APEX2
APLF (C2ORF13)	C2orf13
APTX (aprataxin)	APTX
ATM	ATM
ATR	ATR
ATRIP	ATRIP
BLM	BLM
BRCA1	BRCA1
BRCA2 (FANCD1)	BRCA2
BRIP1 (FANCJ)	BRIP1
BTBD12 (SLX4) (FANCP)	BTBD12
CCNH	CCNH
CDK7	CDK7
CETN2	CETN2
CHAF1A (CAF1)	CHAF1A
CHEK1	CHEK1
CHEK2	CHEK2
CLK2	CLK2
DCLRE1A (SNM1)	DCLRE1A
DCLRE1B (SNM1B)	DCLRE1B
DCLRE1C (Artemis)	DCLRE1C
DDB1	DDB1
DDB2 (XPE)	DDB2
DMC1	DMC1
DUT	DUT
EME1 (MMS4L)	MMS4L, SLX2A, HMMS4, MMS4, FLJ31364

EME2	SLX2B, gs125, FLJ00151
ENDOV	FLJ35220
ERCC1	ERCC1
ERCC2 (XPD)	ERCC2
ERCC3 (XPB)	ERCC3
ERCC4 (XPF)	ERCC4
ERCC5 (XPG)	ERCC5
ERCC6 (CSB)	ERCC6
ERCC8 (CSA)	ERCC8
EXO1 (HEX1)	EXO1
FAAP20 (C1orf86)	FP7162, C1orf86
FAAP24 (C19orf40)	C19orf40
FAN1 (MTMR15)	MTMR15
FANCA	FANCA
FANCB	FANCB
FANCC	FANCC
FANCD2	FANCD2
FANCE	FANCE
FANCF	FANCF
FANCG (XRCC9)	FANCG
FANCI (KIAA1794)	FANCI
FANCL	FANCL
FANCM	FANCM
FEN1 (DNase IV)	FEN1
GEN1	FLJ40869
GIYD1 (SLX1A)	SLX1A, GIYD2
GIYD2 (SLX1B)	SLX1, GIYD2, MGC5178
GTF2H1	GTF2H1
GTF2H2	BTF2P44, BTF2, P44, T-BTF2P44, TFIIH
GTF2H3	GTF2H3
GTF2H4	GTF2H4

GTF2H5 (TTDA)	GTF2H5
H2AFX (H2AX)	H2AFX
HELQ (HEL308)	HEL308
HLTF (SMARCA3)	HLTF
HUS1	HUS1
LIG1	LIG1
LIG3	LIG3
LIG4	LIG4
MAD2L2 (REV7)	MAD2L2
MBD4	MBD4
MDC1	MDC1
MGMT	MGMT
MLH1	MLH1
MLH3	MLH3
MMS19	MMS19
MNAT1	MNAT1
MPG	MPG
MRE11A	MRE11A
MSH2	MSH2
MSH3	MSH3
MSH5	MSH4
MSH6	MSH5
MUS81	MSH6
MUS81	MUS81
MUTYH (MYH)	MUTYH
NBN (NBS1)	NBN
NEIL1	NEIL1
NEIL2	NEIL2
NEIL3	NEIL3
NHEJ1 (XLF, Cernunnos)	NHEJ1
NTHL1 (NTH1)	NTHL1

NUDT1 (MTH1)	NUDT1
OBFC2B (SSB1)	OBFC2B
OGG1	OGG1
PALB2 (FANCN)	PALB2
PARP1 (ADPRT)	PARP1
PARP2 (ADPRTL2)	PARP2
PARP3 (ADPRTL3)	PARP3
PCNA	PCNA
PER1	PER1
PMS1	PMS1
PMS2	PMS2
PMS2L3	PMS2L3
PNKP	PNKP
POLB	POLB
POLD1	POLD1
POLE	POLE
POLG	POLG
POLH	POLH
POLI (RAD30B)	POLI
POLK (DINB1)	POLK
POLL	POLL
POLM	POLM
POLN (POL4P)	POLN
POLQ	POLQ
PRKDC	PRKDC
PRPF19 (PSO4)	PRPF19
RAD1	RAD1
RAD17 (RAD24)	RAD17
RAD18	RAD18
RAD23A	RAD23A
RAD23B	RAD23B

RAD50	RAD50
RAD51	RAD51
RAD51B	RAD51L1
RAD51C (FANCO)	RAD51C
RAD51D	RAD51L3
RAD52	RAD52
RAD54B	RAD54B
RAD54L	RAD54L
RAD9A	RAD9A
RBBP8 (CtIP)	RBBP8
RDM1 (RAD52B)	RDM1
RECQL (RECQ1)	RECQL
RECQL4	RECQL4
RECQL5	RECQL5
REV1L (REV1)	REV1
REV3L (POLZ)	REV3L
RIF1	RIF1
RNF168	RNF168
RNF4	RNF4
RNF8	RNF8
RPA1	RPA1
RPA2	RPA2
RPA3	RPA3
RPA4	RPA4
RRM2B (p53R2)	RRM2B
SETMAR (METNASE)	SETMAR
SHFM1 (DSS1)	SHFM1
SHPRH	SHPRH
SMUG1	SMUG1
SPO11	SPO11
SPRTN (c1orf124)	C1orf124

TDG	TDG
TDP1	TDP1
TDP2 (TTRAP)	TTRAP
TFIIH	BTF2, XPB, RAD25, BTF2, XPBC
TOPBP1	TOPBP1
TP53	TP53
TP53BP1 (53BP1)	TP53BP1
TREX1 (DNase III)	TREX1
TREX2	TREX2
TTDN1 (C7orf11)	MPLKIP, ABHS, C7orf11, ORF20,
TOP3A	TOP3A
TOP3B	TOB3B
UBE2A (RAD6A)	UBE2A
UBE2B (RAD6B)	UBE2B
UBE2N (UBC13)	UBE2N
UBE2V2 (MMS2)	UBE2V2
UNG	UNG
UVSSA (KIAA1530)	KIAA1530)
WRN	WRN
XAB2	XAB2
XPA	XPA
XPC	XPC
XRCC1	XRCC1
XRCC2	XRCC2
XRCC3	XRCC3
XRCC4	XRCC4
XRCC5 (KU80)	XRCC5
XRCC6 (KU70	XRCC6

Supplementary Table S3: Cohort ID and gene expression platform Multicentre (MC)-Adjuvant cohort (n=4640)

Cohort ID	Number (%)
1	497(10.6)
2	286 (6.2)
3	198 (4.3%)
4	200 (4.3%)
5	152 (3.3%)
6	216 (4.7%)
7	203 (4.4%)
8	155 (3.3%)
9	139 (3%)
10	104 (2.2%)
11	556 (12%)
12	357 (7.7%)
13	130 (2.8%)
14	359 (7.7%)
15	266 (5.7%)
16	327 (7.1%)
17	115 (2.5%)
18	58 (1.3%)
19	55 (1.2%)
20	109 (2.4%)
22	158 (2.4%)
**Gene array platform**	**Number (%)**
GPL13667	203 (4.4%)
GPL5049	155 (3.3%)
GPL5345	359 (7.7%)
GPL6098	216 (4.7%)
GPL6486	152 (3.3%)
GPL8300	158 (3.4%)
GPL9128	54 (1.2%)
GPL96	1567(33.8%)
GPL96-GPL	327 (7%)

Supplementary Table S4: Demographics of Multicentre (MC)-Adjuvant cohort (n=4640)

**Characteristics**		**Number**	**% of whole cohort**
Grade			
	1	431	9.3
	2	1290	27.8
	3	1332	28.7
	Data not available	1584	34.2
Lymph node status			
	negative	2015	43.4
	positive	1246	26.9
	Data not available	1379	29.7
ER expression			
	negative	1558	
	positive	2268	
	Data not available	814	
PR expression			
	negative	1441	31.1
	positive	1269	27.3
	Data not available	1930	41.6
HER2 expression			
	negative	1281	27.6
	positive	446	9.6
	Data not available	2913	62.8
ERCC1 expression			
	negative	2359	50.8
	positive	2281	49.2
Relapse status			
	negative	2204	47.5
	positive	967	20.8
	Data not available	1469	31.7
Adjuvant treatment			
	No	1167	25.2
	Yes	1454	31.3
	Data not available	2019	43.5
Chemotherapy			
	No	1906	41.1
	Yes	714	15.4

	Total	2620	56.5
Anthracycline			
	No	1897	40.9
	Yes	338	7.3
	Total	2235	48.2
	Adjuvant Herceptin		
	No	2123	45.8
	Yes	156	3.4
	Total	2279	49.1
Endocrine therapy			
	No	1167	25.2
	Yes	1109	23.9
	Total	2276	49.1

Supplementary Table S5: Multicentre (MC) Neo-Adjuvant cohort (n= 2345)

**Characteristics**		**Number**	**% of whole cohort**
Grade			
	1	-	
	2	579	
	3	726	
	Data not available	999	
ER expression			
	negative	1163	
	positive	1093	
	Data not available	89	
PR expression			
	negative	1182	
	positive	876	
	Data not available	287	
HER2 expression			
	negative	1645	
	positive	518	
	Data not available	182	
ERCC1 expression			
	negative	1180	
	positive	1165	
Relapse status			
	negative	580	
	positive	173	
	Data not available	1592	
Chemotherapy			
	FEC	689	
	FEC-T	1413	
	FEC-T-H	243	
pCR			
	No	1749	
	Yes	596	

Supplementary Table S6: Demographics in TOP trial cohort

**Age (years)**	
**Median, (Range),**	45 (25-70)
**No. of positive lymph nodes**	
Negative	52 (45.6)
Positive	62 (54.4)
Unknown	0 (0-0)
**T stage**	
T0	0 (0-0)
T1 a + b (<10 mm)	16 (14.0)
T1 c (>10-20 mm)	
T2 (>20-50 mm)	79 (69.3)
T3 (>50 mm)	5 (4.4)
T4	14 (12-3)
**Histological grade**	
Low	2 (1.8)
Intermediate	20 (17.5)
High	87 (76.3)
Unknown	5 (4.4)
**Oestrogen-receptor status**	
Positive	0 (0)
Negative	114 (100)
**HER2 overexpression**	
No	87 (76-3)
Yes	27 (23.7)
**Recurrence events**	
No	90 (78.9)
Yes	24 (21.1)
Unknown	0 (0.0)
**Death events**	
Alive, lost follow up or dead from other causes	98 (86.0)
Dead from breast cancer	16 (14.0)
**Follow up survival (months)**	
All cohort: Median (IQR)	33.6 (21.1-46.4)

Supplementary Table S7: Clinicopathological characteristics of Nottingham historical early stage cohort (NUH-ESBC).

Variable	n*	Cases	(%)
**Menopausal status**	1650		
Pre-menopausal		612	(37.0)
postmenopausal		1038	(63.0)
**Tumour Grade (NGS)**	1650		
G1		306	(18.5)
G2		531	(32.2)
G3		813	(49.3)
**Lymph node stage**	1650		
Negative		1056	(64.0)
Positive (1-3 nodes)		486	(29.5)
Positive (>3 nodes)		108	(6.5)
**Tumour size (cm)**	1650		
T1 a + b (≤1.0)		187	(11.0)
T1 c (>1.0 -2.0)		868	(53.0)
T2 (>2.0-5)		579	(35.0)
T3 (>5)		16	(1.0)
**Tumour type**	1650		
IDC-NST		941	(57)
Tubular		349	(21)
ILC		160	(10)
Medullary (typical/atypical)		41	(2.5)
Others		159	(9.5)
**NPI subgroups**	1650		
Excellent PG(2.08-2.40)	Low risk	207	(12.5)
Good PG(2.42-3.40)		331	(20.1)
Moderate I PG(3.42 to 4.4)	High risk	488	(29.6)
Moderate II PG(4.42 to 5.4)		395	(23.9)

Poor PG(5.42 to 6.4)		170	(10.3)
Very poor PG(6.5–6.8)		59	(3.6)
**Survival at 20 years**	1650		
Alive and well		1055	(64.0)
Dead from disease		468	(28.4)
Dead from other causes		127	(7.6)
**Adjuvant systemic therapy (AT**)			
No AT		665	(42.0)
Hormone therapy (HT)		642	(41.0)
Chemotherapy		307	(20.0)
Hormone + chemotherapy		46	(3.0)

* Number of cases for which data were available.

NPI; Nottingham prognostic index, PG; prognostic group

SupplementaryTableS8: Table of antibodies and optimisation conditions used to immunohistochemically profile the Nottingham University Hospitals based cohorts. Detailed below are: Antigens, primary antibodies, clone, source, optimal dilution and scoring system, used for each immunohistochemical marker.

**Antigen**	**Antibody**	**Clone**	**Source**	**Antigen Retrieval**	**Dilution / Incubation Time**	**Distribution**	**Scoring system**	**Cut-offs**
p53	Mouse MAb anti p53	DO7	Novocastra	Citrate pH6	1: 50/ 60 min	Nuclear	% of positive cells	<20% (negative)<br>>20% (High)
ER	Mouse MAb anti-ER-a	SP1	Dako-Cytomation	Citrate pH6	1:150/ 30 min	Nuclear	Allred score	>3 (positive)
ER	Mouse MAb anti-ER-a	EP1	Dako-Cytomation	Citrate pH6	1:80/ 30 min	Nuclear	% positive cells	>1% positive
PR	Mouse MAb anti-PR	PgR636	Dako-Cytomation	Citrate pH6	1:125/ 30 min	Nuclear	% positive cells	>1% positive
CK14	Mouse MAb anti-Ck14	LL002	Novocastra	Citrate pH6	1:40/ 60 min	Cytoplasm	% of positive cells	>10% (positive)
Ck5/6	Mouse MAb anti-Ck5/6	D5/161B4	Dako-Cytomation	EDTApH8	1:100/ 60 min	Cytoplasm	% of positive cells	>10% (positive)
Ck17	Mouse MAb anti-Ck17	E3	Dako-Cytomation	Citrate pH6	1:100/ 60 min	Cytoplasm	% of positive cells	>10% (positive)
Ck18	Mouse MAb anti-Ck18	DC10	Dako-Cytomation	Citrate pH6	1:100/ 60 min	Cytoplasm	% of positive cells	>10% (positive)
HER2	Rabbit antihuman c-erbB2	polyclonal	Dako-Cytomation	None	1:400/ 60 min	Membrane	See text	See text
Ki67	Mouse MAb anti-Ki-67	MIB1	Dako-Cytomation	Citrate pH6	1:300/ 60 min	Nuclear	% of positive cells	0-30% (low)<br>>30% (high)

All sections were pre-treated with microwave antigen retrieval using 0-1% citrate buffer (pH 6) except for HER2 (no pre-treatment). MAb: Monoclonal antibody; ER: oestrogen receptor; PR: progesterone receptor; CK: cytokeratin; HER2 (ERBB2): v-erb-b2 erythroblastic leukemia viral oncogene homolog 2.

Supplemental Table S9: Clinicopathological characteristics of ER- cohort

Variable	n*	Cases	(%)
**Menopausal status**	252		
Pre-menopausal		122	(48.5)
postmenopausal		130	(51.5)
**Tumour Grade (NGS)**	252		
G1		1	(0.3)
G2		27	(10.6)
G3		224	(89.1)
**Lymph node stage**	252		
Negative		121	(48)
Positive (1-3 nodes)		86	(34)
Positive (>3 nodes)		45	(18)
**Tumour size (cm)**	252		
T1 a + b (≤1.0)		28	(11)
T1 c (>1.0 -2.0)		106	(42)
T2 (>2.0-5)		103	(41)
T3 (>5)		15	(6)
**Tumour type**	252		
IDC-NST		224	(89.0)
Tubular		5	(2.0)
ILC		8	(3.0)
Medullary (typical/atypical)		5	(2.0)
Others		0	(4.0)
**NPI subgroups**	252		
Excellent PG(2.08-2.40)	Low risk	0	(0.0)
Good PG(2.42-3.40)		0	(0.0)
Moderate I PG(3.42 to 4.4)	High risk	111	(44.0)
Moderate II PG(4.42 to 5.4)		81	(32.0)

Poor PG(5.42 to 6.4)		38	(15.0)
Very poor PG(6.5–6.8)		22	(9.0)
**Survival at 5 years**	252		
Alive and well		176	(70.0)
Dead from disease		73	(29.0)
Dead from other causes		3	(1.0)

* Number of cases for which data were available.

NPI; Nottingham prognostic index, PG; prognostic group

Supplementary Table S10: Multivariate backward step-wise Cox analysis for DNA repair genes associated with Overall Survival (OS) in ER+ METABRIC cohort.

Gene	HR	95% CI	P value
ERCC1	0.578893	0.35-0.96	0.032364
EXO1	1.333204	1.06-1.67	0.013043
FEN1	1.377967	1.11-1.71	0.003992
HLTF	1.584208	1.27-1.98	0.000048
PMS2	0.599729	0.44-0.83	0.001791
RBBP8	0.71	0.62-0.81	0.000001
TDG	1.33	1.08-1.65	0.007515

Supplementary-Table-S11. Clinicopathological significance of ERCC1 protein expression in breast cancers.

	**ERCC1 protein level**		**P- value**	***P -Value** (Adjusted)
	Low	High		
**A) Pathological Parameters**				
**Tumour Size**				
≤1cm	47 (8.5%)	42 (9.6%)	0.073	0.0892
>1-2cm	267(48.4%)	219(49.9%)		
>2-5cm	211(38.2%)	170(38.7%)		
>5cm	27 (4.9%)	8 (1.8%)		
**Tumour Grade**				
1	48(8.6%)	61(13.9%)	**$1.33x10^{-4}$**	**<0.00001**
2	129(23.2%)	135(30.7%)		
3	380(68.2%)	244(55.5%)		
**Lymph node status**				
Negative	350(62.7%)	279(63.4%)	0.975	10.7250
1-3 LN positive	156(28.0%)	121(27.5%)		
> 4 LN positive	52 (9.3%)	40 (9.1%)		
**NPI**				
≤ 3.4	101(18.1%)	124(28.2%)	**$1.639x10^{-4}$**	**<0.00001**
>3.4	456(81.9%)	316(71.8%)		
**Mitotic Index**				
1 (low; mitoses)	126(22.8%)	136(31.1%)	**0.001**	**0.0022**
2 (medium; mitoses)	93 (16.8%)	88 (20.1%)		
3 (high; mitosis)	334(60.4%)	214(48.9%)		
**Tubule Formation**				
1 (>75% definite tubule)	18 (3.3%)	22(5.0%)	**0.044**	0.0605
2 (10%-75% definite tubule)	142(25.7%)	136(31.1%)		
3 (<10% definite tubule)	393(71.1%)	280(63.9%)		
**Pleomorphism**				
1 (small-regular uniform)	8 (1.4%)	11(2.5%)	**0.002**	**0.0037**
2 (Moderate variation)	117(21.2%)	132(30.1%)		
3 (Marked variation)	428(77.4%)	295(67.4%)		
**Tumour Type**				
IDC-NST	371(73.9%)	263(65.3%)	**0.007**	**0.0110**
Tubular Carcinoma	65 (12.9%)	69 (17.1%)		
Medullary Carcinoma	17 (3.4%)	7 (1.7%)		
ILC	19 (3.8%)	27 (6.7%)		
Others	30 (6.0%)	37 (9.2%)		
**ER expression**				
Negative	471(86.6%)	381(87.6%)	**0.000001**	**<0.00001**
Positive	73 (13.4%)	54 (12.4%)		
**Her2 overexpression**				
Negative	471(86.6%)	381(87.6%)	0.642	0.7062
Positive	73 (13.4%)	54 (12.4%)		
**Basal like phenotype**				
Negative	361(69.6%)	334(79.9%)	**$3.2x10^{-4}$**	**<0.00001**
Positive	158(30.4%)	84 (20.1%)		
**Triple negative phenotype**				
Negative	314(57.9%)	316(72.8%)	**$1.377x10^{-6}$**	**<0.00001**
Positive	228(42.1%)	118(27.2%)		

Supplementary Table S12: Multivariate Cox regression analysis for breast cancer specific survival (BCSS) at 20 years follow up in Nottingham series including interaction terms.

Variables	HR	95.0% CI		P value
		Lower	Upper	
**ERCC1 (+)**	0.69	0.49	0.97	**0.035***
**ER (+)**	1.59	0.94	2.66	0.082
**PR (+)**	0.76	0.51	1.15	0.194
**HER2 (+)**	1.16	0.95	1.41	0.137
**Bcl2 (+)**	0.42	0.30	0.61	**<0.0001**
**Tumour Size (continuous)**	1.22	1.001	1.26	**0.048***
**Lymph node (LN) status**				**<0.0001**
Negative	1			
1-3 positive LNs	2.05	1.45	2.91	
>3 positive LNs	4.45	2.85	6.97	
**Histological grade**				**<0.001***
Low	1			
Intermediate	1.36	0.66	2.79	
High	2.65	1.34	5.25	
**Hormone therapy**	1.09	0.61	1.95	0.767
**Chemotherapy (CMF)**	1.17	0.79	1.72	0.444
**Interaction term** **Chemotherapy* ERCC1**	2.42	1.14	5.13	**0.022***
**Interaction term** **Hormone therapy* Oestrogen receptors**	0.88	0.44	1.75	0.704

*Statistically significant at p<0.05

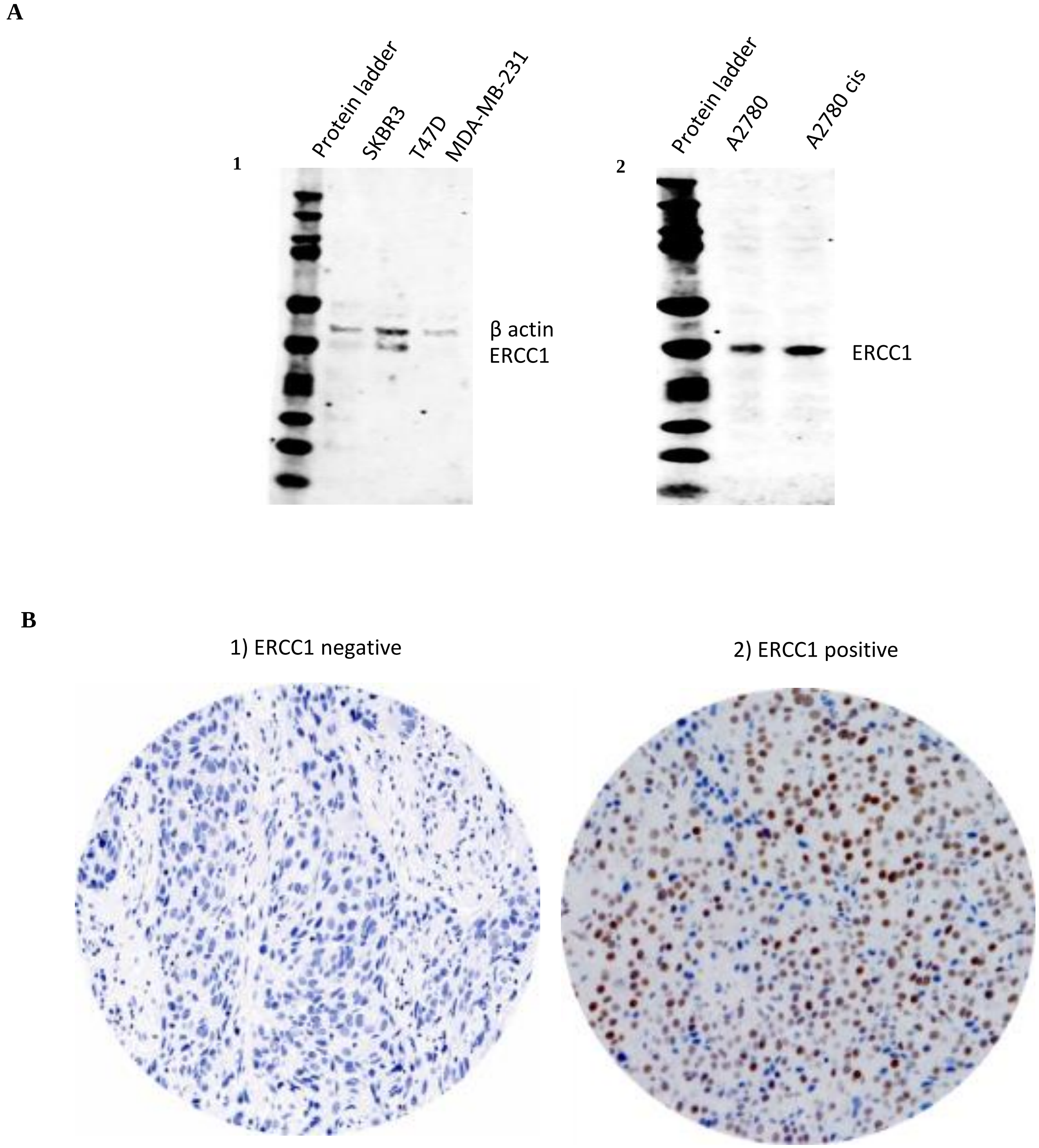

Supplementary Figure S1

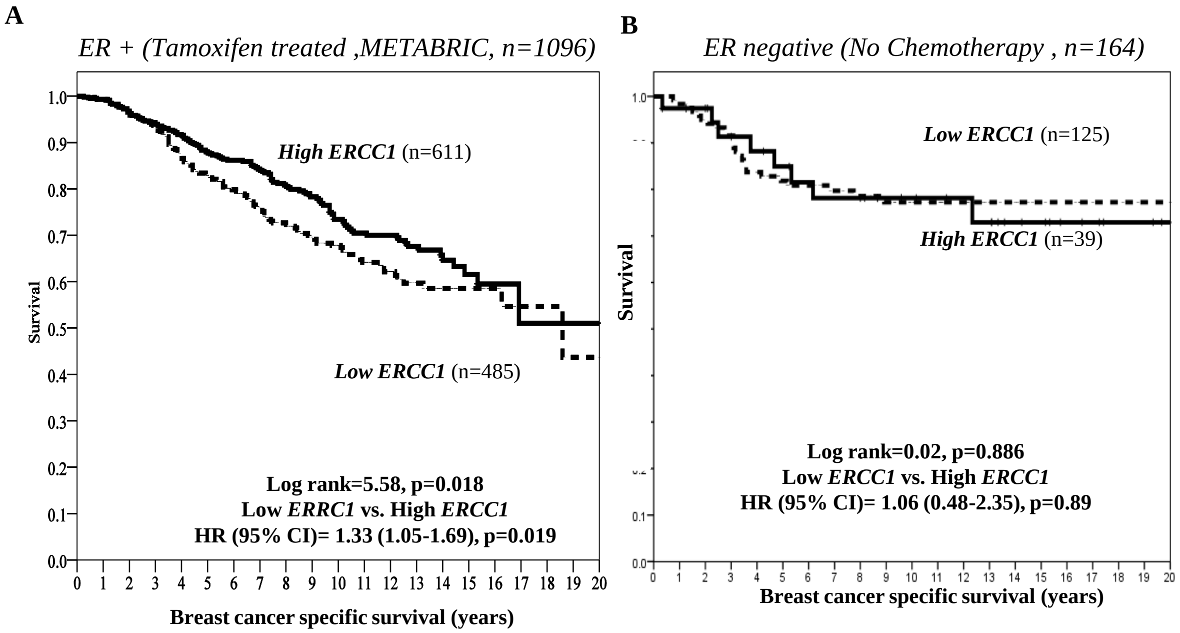

Supplementary Figure S2
